# Unravelling the Links between Phage Adsorption and Successful Infection in *Clostridium difficile*

**DOI:** 10.3390/v10080411

**Published:** 2018-08-06

**Authors:** Anisha Mahendra Thanki, Grace Taylor-Joyce, Ahmed Dowah, Janet Yakubu Nale, Danish Malik, Martha Rebecca Jane Clokie

**Affiliations:** 1 Department of Infection, Immunity and Inflammation, University of Leicester, Leicester LE1 7RH, UK; asad2@le.ac.uk (A.D.); jn142@le.ac.uk (J.Y.N.); 2 School of Life Sciences, Warwick University, Warwick CV4 7AL, UK; g.taylor-joyce@warwick.ac.uk; 3 Chemical Engineering Department, Loughborough University, Loughborough LE11 3TU, UK; d.j.malik@lboro.ac.uk

**Keywords:** *Clostridium difficile*, phage adsorption, phage tail fibers, phage-host interactions

## Abstract

Bacteriophage (phage) therapy is a promising alternative to antibiotics for the treatment of bacterial pathogens, including *Clostridium difficile*. However, as for many species, in *C. difficile* the physical interactions between phages and bacterial cells have not been studied in detail. The initial interaction, known as phage adsorption, is initiated by the reversible attachment of phage tail fibers to bacterial cell surface receptors followed by an irreversible binding step. Therefore binding can dictate which strains are infected by the phage. In this study, we investigated the adsorption rates and irreversible binding of three *C. difficile* myoviruses: CDHM1, CDHM3 and CDHM6 to ten strains that represent ten prevalent *C. difficile* ribotypes, regardless of their ability to infect. CDHM1 and CDHM3 phage particles adsorbed by ~75% to some strains that they infected. The infection dynamics for CDHM6 are less clear and ~30% of the phage particles bound to all strains, irrespective of whether a successful infection was established. The data highlighted adsorption is phage-host specific. However, it was consistently observed that irreversible binding had to be above 80% for successful infection, which was also noted for another two *C. difficile* myoviruses. Furthermore, to understand if there is a relationship between infection, adsorption and phage tail fibers, the putative tail fiber protein sequences of CDHM1, CDHM3 and CDHM6 were compared. The putative tail fiber protein sequence of CDHM1 shares 45% homology at the amino acid level to CDHM3 and CDHM6, which are identical to each other. However, CDHM3 and CDHM6 display differences in adsorption, which highlights that there is no obvious relationship between putative tail fiber sequence and adsorption. The importance of adsorption and binding to successful infection is often overlooked, and this study provides useful insights into host-pathogen interactions within this phage-pathogen system.

## 1. Introduction

*Clostridium difficile* is the most common cause of infectious antibiotic-associated diarrhea in the developed world [1,2]. *C. difficile* infection (CDI) predominately stems from the administration of broad spectrum antibiotics, which causes dysbiosis, allows *C. difficile* to colonize the gut and cause disease [3]. Patients can suffer from mild diarrhea to pseudomembranous colitis to death in severe infections and high rates of 20% disease relapse are observed following antibiotic therapy [4,5,6]. To add to the burden, over 350 different subgroups have been identified, which are grouped into ribotypes based on their sequences between 16S and 23S rRNA genes [7,8]. The most prevalent *C. difficile* ribotypes worldwide associated with infection are 002, 005, 014/020, 015 and the hypervirulent ribotypes 027 and 078 [9]. Only three antibiotics (metronidazole, vancomycin and fidaxomicin) are available to treat CDI, and worryingly *C. difficile* is beginning to develop resistance against them [10,11]. Consequently, replacements or adjuncts to antibiotics are needed to treat CDI. Bacteriophages (phages) are viruses that target and kill bacteria with high specificity to the level of individual bacterial species and even strains, and they have great promise to be developed as alternative therapeutics for CDI [12,13].

Isolating phages that lyse clinically prevalent *C. difficile* ribotypes has proven to be challenging, in part due to the difficulties with working with an anaerobic organism [14,15]. However significant progress has been made, and phages have been isolated that can lyse clinically relevant ribotypes [16,17,18,19,20,21,22] and are specific so they do not infect other Clostridia species or hospital pathogens, such as *Pseudomonas aeruginosa* [23]. To develop a phage-based product to target multiple *C. difficile* ribotypes, individual and combinations of seven isolated phages: CDHM1–6 and CDHS1 were tested. CDHM3 was identified as the broadest phage from the collection and was able to infect 31/80 strains from 12 different ribotypes, which included all seven strains of ribotype 014/020 screened. However, significant bacterial lysis was observed with a four-phage cocktail (CDHM1, 2, 5, 6) that was able to completely eradicate *C. difficile* in vitro [15]. The same four-phage cocktail could significantly reduce *C. difficile* colonization in vivo using hamster and in *Galleria mellonella* larva models [15,23,24,25]. Similarly, in vitro batch fermentation human colon models and human cell lines [26] have provided useful insights in to the specificity of *C. difficile* phages and shown phages do not have a deleterious impact on commensal gut bacteria [27,28,29].

Although we now have over 10 *C. difficile* phages in our collection, little is known about their physical interactions with their bacterial host. Understanding the initial phage-host interaction is an important step towards determining infection parameters, which could help answer if phages can bind to cells they cannot infect and if so is bacterial resistance at this level [30]. Adsorption and successful attachment can then potentially be exploited to broaden the infectivity of phages and to develop innovative biotechnological phage-based tools for bacterial diagnostics [31,32].

Phage binding to bacterial cells occurs when the phage receptor-binding proteins or tail fibers, located at the tip of the tail, bind to their target receptor(s) on the bacterial cell [33]. The attachment between the phage tail fibers and bacterial receptors is highly specific and even within a single bacterial species, multiple phage receptors are generally present [34,35]. Consequently, tails fibers play a vital role in determining the host range and in part dictate which bacterial strains are lysed by the phage [29]. Phage adsorption occurs by a three-step process of initial contact, reversible attachment and irreversible binding [32]. The first step involves random phage collision governed by Brownian motion or diffusion and once adsorbed the phages undergo a “random walk” on the bacterial cell surface until it is captured by the phage receptor [33]. Then phages bind reversibly to their receptor on the bacterial surface and can still become de-absorbed, a process which has been experimentally shown to keep the phage close to its specific receptor [36]. The final step is permanent as phages bind irreversibly either to the same receptor where they were reversibly bound to, or to a second receptor [29]. Irreversible phage binding causes conformational rearrangement of phage structures, generally the baseplate, which in turn leads to the insertion of viral DNA into the bacterial host [30]. Consequently, successfully reversible and subsequent irreversible phage binding is necessary for phage infection.

Extensive adsorption studies have been conducted for phages infecting Gram-positive bacteria including: *Bacillus subtilis*, *Listeria monocytogenes* and *Lactococcus lactis* [37,38,39,40,41]. However, studies on reversible and irreversible adsorption of *C. difficile* phages to clinically relevant strains they can and cannot infect have not been conducted. In this study, we determined the adsorption dynamics of three *C. difficile* myoviruses: CDHM1, CDHM3 and CDHM6 as we hypothesize there is a link between phage binding and infection. These three phages were selected of the basis that they have similar host ranges; CDHM1 and CDHM6 are part of four-phage cocktail previously shown to effectively lyse *C. difficile*: and all three phages can all be amplified on the same *C. difficile* strain [13,22,25]. Adsorption of all three phages to ten different clinically relevant *C. difficile* strains, regardless of whether they could infect the strain or not was investigated [15]. The specificity of the phages was determined by also assessing their adsorption dynamics on strains of *Escherichia coli*, Methicillin-resistant *Staphylococcus aureus* (MRSA) and *Pseudomonas aeruginosa*. In addition, irreversible adsorption was analyzed for all strain-phage combinations to determine whether below ~40% irreversible phage adsorption correlated with the inability of the phage to infect that particular strain. Furthermore, to determine if host range, phage adsorption and irreversible binding is generalized for *C. difficile* phages, binding was investigated for further two *C. difficile* phages. Additionally, to understand if there is a link between adsorption, infection and putative tail fiber protein sequence, these protein sequences of CDHM1, CDHM3 and CDHM6 were compared against each other, and to other previously isolated *C. difficile* phages. Our data suggests that although there is no link between *C. difficile* phage infection, adsorption and putative tail fiber protein sequence, there is a link between irreversible phage binding and infection.

## 2. Materials and Methods

### 2.1. Bacterial Strains and Phages Used and Their Growth Conditions

The ten *C. difficile* strains used in this study were AKC (ribotype 002), AIN (ribotype 005), ASH (ribotype 013), ATJ (ribotype 014/020), ARU (ribotype 026), CD105LC1 (ribotype 027), CD105HE1 (ribotype 076), ASS (ribotype 078), APT (ribotype 087) and ARZ (ribotype 107). All *C. difficile* strains were from our laboratory collection. All strains, except CD105HE1 were isolated from clinical fecal samples from patients who tested positive for *C. difficile* toxins, strains were ribotyped in our laboratory as previously described [40]. Strain CD105HE1 was isolated from the environment [42,43]. All strains were stored in 25% glycerol at −80 °C and routinely cultured on 1% brain heart infusion (BHI; Oxoid, Basingstoke. UK) supplemented with 7% defibrinated horse blood (TCS Biosciences Ltd., Buckingham, UK) and incubated anaerobically (10% H_2_, 5% CO_2_ and 85% N_2_, MinMACS, Don Whitley Scientific, Bingley, UK). To prepare liquid cultures single colonies were inoculated into fastidious anaerobic broth (FAB; BioConnections, Kynpersley, UK) and stored in the anaerobic chamber at 37 °C.

*Escherichia coli* and MRSA strains were isolated in our laboratory and the *Pseudomonas aeruginosa* PAO1 ATCC 15692 strain were used in this study [43]. All strains were stored as 50% glycerol stocks at −80 °C and routinely grown on LB 1% agar plates incubated overnight at 37 °C. To prepare liquid cultures a single colony was taken from plates and inoculated into 5 mL LB broth. Cultures were grown overnight at 37 °C with shaking at 100 rpm.

The *C. difficile* myoviruses CDHM1, CDHM2, CDHM3, CDHM5 and CDHM6 were isolated in our laboratory and have previously been described in detail [15,25,28,42]. All phages were individually propagated to 10^9^ plaque forming units (PFU)/mL in broth cultures of strain CD105HE1 (will be referred to as propagation host), filtered using 0.22 μL filters (Merck Millipore Ltd., Nottingham, UK) and stored at 4 °C until use.

### 2.2. Phage Host Range Analysis and Efficiency of Plating

The phages were screened for lytic activity on *C. difficile* strains using the spot testing method [15,44]. Briefly, 300 μL of an overnight *C. difficile* liquid culture grown in FAB was mixed with 3 mL of 0.5% BHI agar overlay with 0.01 M CaCl_2_, 0.4 M MgCl_2_ (will be referred to as salts) and poured onto BHI 1% agar circular 90 mm plates. To the agar overlay 10 μL of phage lysate at titers of ~5 × 10^8^ PFU/mL were spotted, incubated under anaerobic conditions overnight at 37 °C and plates were assessed for lytic activity.

Phages were tested for lytic activity on *E. coli*, *P. aeruginosa* and MRSA using the spot testing method [15,44]. Overnight liquid cultures of the bacteria grown in LB broth were mixed with 3 mL LB 0.5% agar and poured onto LB 1% agar plates, to which 10 μL of phage lysate were spotted. Plates were incubated at 37 °C overnight and assessed for lytic activity.

Efficiency of plating (EOP) was assessed using a previously described method [44,45]. Briefly a series of 10-fold dilutions were made for each phage and 10 μL of the dilutions were spotted on lawns of *C. difficile*. Average PFU/mL values were calculated from three biological replicates, each with three technical repeats, which were then compared against the EOP of the phages on their propagation host CD105E1 and are presented as percentage differences.

### 2.3. Phage Adsorption and Irreversible Phage Binding

Liquid cultures of *C. difficile* in BHI broth; and *E. coli*, *P. aeruginosa* and MRSA in LB broth were incubated overnight and in the morning were diluted 100-fold in 1 mL of fresh media. *C. difficile* cultures were incubated anaerobically till an optical density (OD_550_) of 0.2 was reached and *E. coli*, *P. aeruginosa* and MRSA cultures were incubated at 37 °C with shaking at 100 rpm till the cultures were at OD_600_ of 0.2. At these OD readings, bacterial counts were ~4 × 10^7^ colony forming units (CFU)/mL, which was previously determined by growth curves. At this OD (reflective of exponential growth), the phage being tested was added at a multiplicity of infection of 1. In addition 1 mL of salt solution (0.01 M CaCl_2_, 0.4 M MgCl_2_) was added, as it significantly improved phage adsorption (Supplementary Figure S1) and is consistent with the literature [33,35,46]. The suspension was mixed by gentle inversion and incubated for 30 min in conditions specific for the bacterial species being examined.

A 30 min incubation time was selected as the optimal sample point to quantify the number of total phages adsorbed, as at 40 min new phage progeny were released for all phages on their propagation host. A phage-only control was also included to determine if there are interactions between the phage and the media; if the phages were being degraded by experimental procedure and to confirm the adsorption data collected were not experimental artifacts. After the 30 min incubation period samples were centrifuged at 21,000× *g* for 10 min at 4 °C to pellet phage-adsorbed cells. The supernatant contained free, unabsorbed phages and will be referred to as S1. The supernatant was serially diluted 10-fold in BHI broth and spot-tested on a lawn of their host, CD105E1 to determine phage titers [44]. The percentage of total phage particles adsorbed was calculated by comparing phage titers in the phage-only control supernatant to the number of free phages in the supernatant of the different bacterial-phage suspensions. Percentages were plotted on a bar graph using the software GraphPad Prism version 7 [47] and average data was plotted from three biological repeats, each three technical replicates.

To determine what proportion of phages had irreversibly bound to the pellet the phage-adsorbed cells were re-suspended in 1 mL of BHI broth and vortexed to remove reversibly bound phage particles. The samples were centrifuged (21,000× *g* for 10 min at 4 °C) to re-pellet the bacteria. The supernatant was then removed, serially diluted 10-fold and spot-tested to determine phage titers. The supernatant contained reversibly bound phage and will be referred to as S2.

To calculate total bound phage and the percentage of irreversibly bound phage, the following three formulae were used:

(i) Phage titer in control sample − S1 = total bound phage
(ii) S2 × 100/total bound phage = percentage reversibly bound phage
(iii) 100 − percentage reversibly bound phage = percentage of irreversibly bound phage.

### 2.4. Alignment and Phylogenetic Analysis

The *C. difficile* putative tail fiber sequence of CDHM1 (locus ID: YP_009032171.1) is available on NCBI and putative tail fiber sequences of CDHM3 (locus ID: MH256665) and CDHM6 (locus ID: MH256666) have been deposited. For each phage, to date only one protein has been annotated as the putative tail fiber protein. A further 15 putative tail fiber protein sequences of other *C. difficile* myoviruses were obtained from Genbank, their accession numbers are listed in Supplementary Table S1.

Protein sequence alignment and phylogenetic analysis of tail fiber proteins were conducted using the program Molecular Evolutionary Genetics Analysis (MEGA) package, version 7 (Pennsylvania State University, Pennsylvania, USA). Phylogenetic analysis of *C. difficile* tail fiber proteins was conducted using the Neighbor-joining (NJ) method and bootstrapped with 500 replicates.

### 2.5. Statistical Analysis

Experimental data obtained from three independent biological replicates, each with three technical replicates were analyzed with the two-tailed student’s t-test. Results were significant if *p* values were ≤ 0.05.

## 3. Results

### 3.1. Phage Host Range and Effeciency of Plating

The lytic activity of phages CDHM1, CDHM3 and CDHM6 was assessed on ten bacterial strains that are representatives of ten clinically relevant *C. difficile* ribotypes worldwide (Table 1). All phages could lytically infect strain ATJ (ribotype 014/020) and their propagation host (as previously described [15]). On some strains the infection appeared to be incomplete, for example phages CDHM1 and CDHM6 produced turbid, hazy clearings on strain ARU (ribotype 026), which indicates that it is not a true lytic infection. Similarly, CDHM3 produced turbid clearings on CD105LC1 (ribotype 027) and ASH (ribotype 013). All three phages were unable to infect representative *C. difficile* strains from ribotypes 002, 005, 013, 078, 087 (as previously described [15]) and 107. As expected the phages could not lyse the *E. coli*, *P. aeruginosa* and MRSA strains examined. It should be noted here that all three phages are able to lyse more *C. difficile* strains but for this study we only focused on strains that represent clinically relevant ribotypes worldwide [15].

To explore both the adsorption dynamics and probe the efficiency of the phages to infect different strains, EOP studies were conducted. The EOP of CDHM1 on strain ATJ (ribotype 014/020) was not significantly different to the EOP on its propagation host (*p* > 0.05). In contrast there was ~20% reduction in phage CDHM3 and CDHM6 titers on strain ATJ when compared to their propagation host CD105HE1. On strains where only turbid clearing was observed from the host range analysis, hazy clearings were observed until 10^−2^ dilution of the phage lysate stocks. At higher dilutions no clearing or plaques were present, which suggests that the phages are unable to replicate in these strains and that the observed clearing could be caused by another mechanism, such as lysis from without.

### 3.2. Adsorption of CDHM1, CDHM3 and CDHM6 to Relevent C. difficile Ribotypes and Other Pathogens

#### 3.2.1. Adsorption Dynamics of CDHM1

**Figure 1:**
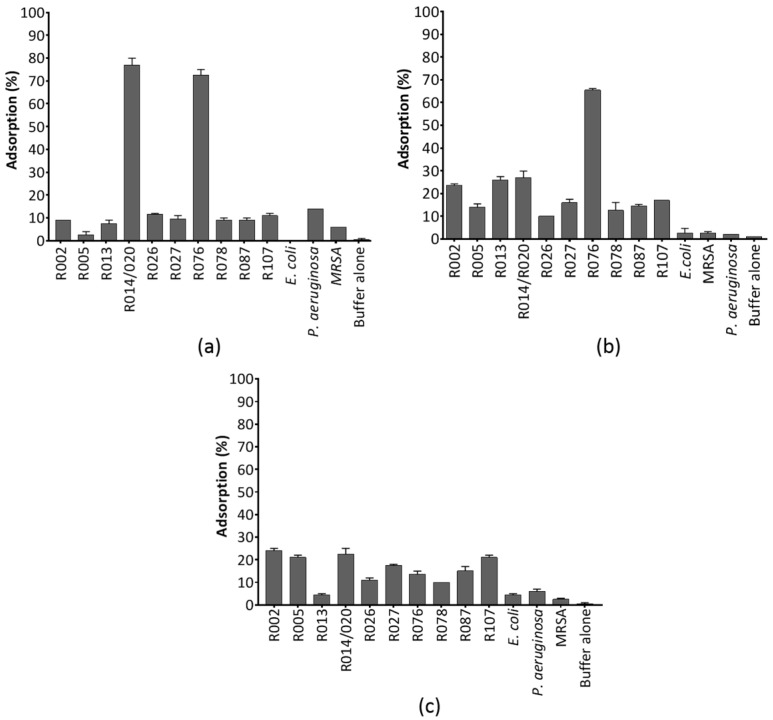
The adsorption of phages CDHM1 (**a**), CDHM3 (**b**) and CDHM6 (**c**) to ten representative isolates of clinically relevant *C. difficile* ribotypes and representative *E. coli*, *P. aeruginosa* and MRSA strains. A phage-only control in buffer alone was included. Bar graphs show phage adsorption after 30 min. Bars represent average percentage adsorption from three independent experiments, each with three technical repeats and error bars represent standard error of the mean.

Within 30 min over 75% of CDHM1 had adsorbed to strains ATJ (ribotype 014/020) and CD105HE1 (ribotype 076), which were both strains the phage can infect and has a high EOP on (Figure 1a and Supplementary Figure S2a). These results suggest that a high proportion of phage binding correlates with successful infection and subsequent replication of CDHM1. In contrast ~15% of CDHM1 particles bound to strain ARU (ribotype 026), on which turbid clearing was observed from the host range analysis (Table 1). For the remaining seven bacterial strains, which were not infected by the phage, there was ~15% adsorption. Also, ~15% phage particles bound to the *P. aeruginosa* strain. CDHM1 bound by ~5% to *E. coli* and MRSA strains, comparable to the phage control.

#### 3.2.2. Adsorption Dynamics of CDHM3

Approximately 70% of phage CDHM3 particles bound to its propagation host (CD105HE1) on which it clearly undergoes a lytic infection (Figure 1b and Supplementary Figure S2c). The dynamics with other strains are a little more complex as ~28% particles adsorbed to strain ATJ, which CDHM3 infects with a high EOP, and ASH (ribotype 013), a strain that CDHM3 can only partially clear (Table 1). This result suggests adsorption dynamics for a given phage may differ for different host-phage combinations and a high adsorption percentage does not always result in a lytic infection. CDHM3 also produced turbid clearing on the CD105LC1 (ribotype O27) strain and phage binding was ~18%. Similarly, ~18% of phages bound to APT (ribotype 087) and ARZ (ribotype 107), which were both strains the phage could not infect. CDHM3 did not display non-specific binding to *E. coli*, *P. aeruginosa* and MRSA strains as binding values were equivalent to the phage-only control.

#### 3.2.3. Adsorption Dynamics of CDHM6

Adsorption of CDHM6 did not follow the patterns observed with phages CDHM1 or with CDHM3 (Figure 1c and Supplementary Figure S2e). It was observed that 3–30% of phage CDHM6 were adsorbed to all *C. difficile*, *E. coli*, *P. aeruginosa* and MRSA strains, regardless of whether the phage was able to sustain a lytic infection on the strain. Interestingly, CDHM6 bound by less than 20% to its propagation host and by ~22% to strain ATJ, which are both strains the phages can infect. CDHM6 had the highest adsorption percentage at ~30% to AKC (ribotype 002), which is a strain the phage does not infect. CDHM6 partially cleared the ribotype 026 strain and ~13% phage adsorbed to the cells, which was the same binding percentage noted for strain ASS (ribotype 078) that was not infected by the phage. On *E. coli*, *P. aeruginosa* and MRSA strains the phage bound by less than 5%.

### 3.3. Irreversible Binding of Phages CDHM1, CDHM3 and CDHM6 to Different C. difficile Ribotypes

Phages CDHM1, CDHM3 and CDHM6 revealed differences in adsorption dynamics between different phage-strain combinations. To further probe the adsorption dynamics of phage-strain combinations, we compared the percentage of irreversibly bound phages to total bound phages (Table 2) for all *C. difficile* strains used in the adsorption studies (Figure 1). The data suggests that there was a distinct correlation between high irreversible phage binding from the total phages bound (includes both reversible and irreversible bound phages) and infection. This is because >85% of irreversible binding was noted for all phages to strains the phages could infect, regardless of whether the total phage bound was 20% or 75%. Additionally, on strains where turbid clearing was observed, 50–60% phages irreversibly bound. For example, CDHM1 and CDHM6 irreversibly bound to the ribotype 026 strain by ~60% and ~58% respectively from the total phage bound. On *C. difficile* strains not infected by the phages irreversible binding was consistently below 15%, indicating 85% of phages were reversibly bound from the total phage bound. The two exceptions were irreversible binding of CDHM1 to strain APT by an average 29% and CDHM6 to strain ASH by 21%.

The irreversible binding protocol involved a step where bacterial-phage samples were vortexed to remove reversibly bound phages but vortexing could potentially structurally damage the phage, which can lead to a reduction in phage titers. To determine if this occurred phage-only controls were included in the study. Results highlighted vortexing did not cause a significant drop in phage titers for all phages used in this study (Supplementary Figure S2).

### 3.4. Host Range, EOP, Adsorption and Irreversible Binding of Other C. difficile Phages to Different Ribotypes

Adsorption dynamics and irreversible phage binding were investigated for two more *C. difficile* myoviruses: CDHM2 and CDHM5 to all the clinically relevant strains used in this study. This was to determine whether the adsorption dynamics and patterns observed for irreversible binding are generalized for *C. difficile* phages (Table 3). Both phages CDHM2 and CDHM5 were selected as they are part of the four-phage cocktail, previously shown to lyse *C. difficile* in vitro and in vivo [15,17,25,26,27] and they share the same propagation host as CDHM1, CDHM3 and CDHM6.

CDHM2 and CDHM5 were only able to infect strains ATJ and CD105HE1, with high EOP at 95% and 100% respectively. The adsorption profiles of phages CDHM2 (Supplementary Figure S2b) and CDHM5 (Supplementary Figure S2d) were very similar to CDHM1 as they bound by high percentages to strains they could infect. CDHM2 bound by high adsorption efficiencies to ATJ by ~82% and to CD105HE1 by ~79% and CDHM5 adsorbed to ATJ and CD105HE1 by ~82% and ~80% respectively. For all other isolates CDHM2 and CDHM5 could not infect, adsorption ranged from 2–37%, similar to CDHM3 and CDHM6. Therefore, the absorption data highlights and supports the observation that adsorption efficiencies vary for different *C. difficile* host-phage combinations and no generalized trends were seen.

However, the irreversible phage binding data for CDHM2 and CDHM5 further supports that there is a strong correlation between high irreversible binding and infection as both phages irreversibly bound to strains ATJ and T6 by over 90% (Table 3). This was consistently observed for CDHM1, CDHM3 and CDHM6, which could suggest that high irreversible binding and infection is generalized for *C. difficile* myoviruses.

### 3.5. Alignement of Putative Phage Tail Fibers

**Figure 2:**
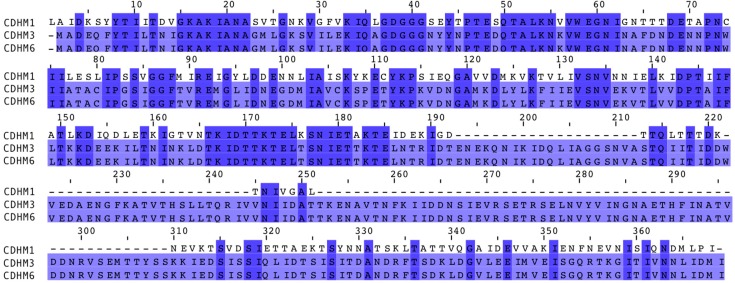
Alignment of putative tail fiber protein sequences of *C. difficile* phages CDHM1, CDHM3 and CDHM6. The alignment tool MUSCLE was used and gaps in the sequence are indicated with dashed lines and numbers above mark the amino acid number. The length of each sequence was 267, 368 and 368 amino acids for phages CDHM1, CDHM3 and CDHM6 respectively. The color of the amino acid represents its percentage identity to the consensus sequence, where the darker the color the higher the percentage identity.

To understand the adsorption dynamics of CDHM1, CDHM3 and CDHM6 phages at a genetic level, the sequences of each putative tail fiber protein were analyzed. Previous studies of well-characterized phages that infect Gram-positive bacteria have experimentally shown that their phage tail fibers play an important role in phage adsorption and binding to the bacterial cell receptor to initiate infection. The genes encoding tail fibers are located downstream of the tape measure protein and upstream of the holin protein [48,49]. Taking this into account, the location of the putative tail fiber proteins annotated within the CDHM1, CDHM3 and CDHM6 phage genomes were also located in the same position in their genomes. The putative tail fiber protein sequences of CDHM1, CDHM3 and CDHM6 were aligned and compared to each other (Figure 2). Despite the differences in phage adsorption the putative tail fiber protein sequences of phages CDHM3 and CDHM6 were 100% identical. However, the CDHM1 putative tail fiber protein only shared 45% sequence homology with both phages. In addition, phage CDHM1 putative tail fiber protein sequence is much shorter in amino acid length compared to CDHM3 and CDHM6. The alignment shows that most of the similarities between the CDHM1 sequence and the other two putative tail fiber proteins are towards the N-terminus, very little similarity is seen at the C-terminus.

### 3.6. Phylogentic Analysis of Putative Tail Fiber Proteins of C. diffiicle Phages

**Figure 3:**
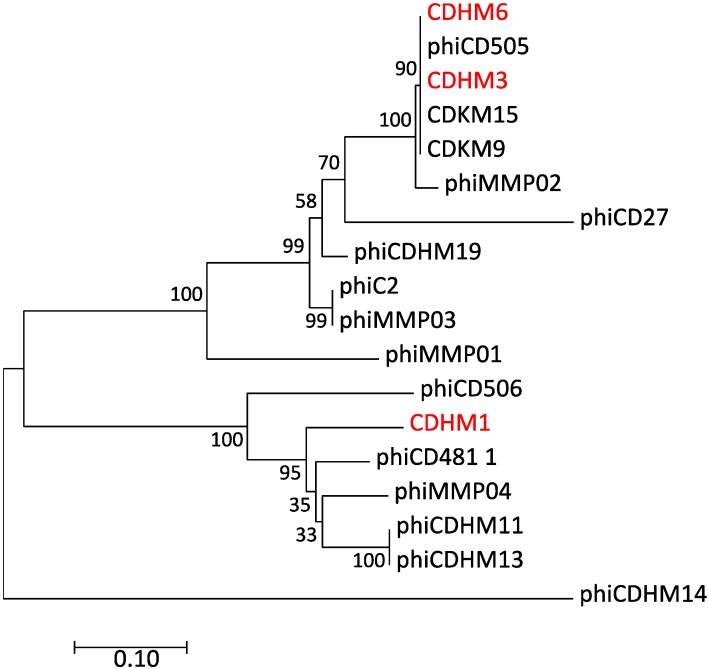
Evolutionary relationship of putative tail fiber proteins of *C. difficile* phages was constructed using the Neighbor-Joining method. The bootstrap consensus tree was inferred from 500 replicates was taken to represent the evolutionary history of *C. difficile* phage putative tail fiber proteins and bootstrap values are shown next to the branches. The tree is drawn to scale and the scale bar represents the relative genetic distances. The evolutionary distances were computed using the p-distance method. All positions that contained gaps and missing data were eliminated from the analysis. The analysis involved 18 protein sequences, which included 15 annotated *C. difficile* myovirus tail fiber proteins available on NCBI (listed in Supplementary Table S1) and myovirus CDHM1, CDHM3 and CDHM6 from the present study (highlighted in red). Evolutionary analyses were conducted in MEGA7.

To analyze the evolutionary relationship of the putative tail fiber protein sequences of CDHM1, CDHM3 and CDHM6, they were compared to 15 other *C. difficile* myoviruses. The 18 putative tail fiber protein sequences were used to construct a phylogenetic tree using the NJ method (Figure 3 and Supplementary Table S1). Only *C. difficile* myoviruses were used in this study as CDHM1, CDHM3 and CDHM6 all belong to Myoviridae family. Furthermore, previous phylogenetic analyses of *C. difficile* siphoviruses based on their protein sequences showed they are very diverse [15,21]. Two distinct clades were identified, and the three phages discussed in the present study were all part of clade 1. Phages CDHM3 and CDHM6 grouped together into one tight subclade with *C. difficile* phages CDHM15, CDKM9 and phiCD505, with 90% bootstrap value. The putative tail fiber protein sequence of CDHM1 was more diverse and was part of a different subclade. Unlike the putative tail fiber proteins of CDHM3 and CDHM6, CDHM1 did not form a tight subclade with other *C. difficile* phages. However, CDHM1 was most closely related to a cluster of four *C. difficile* phages: phiCD48.1, phiMMP04, phiCDHM11 and phiCDHM13. Clade 2 only included *C. difficile* phage phiCDHM14, isolated in a previous study.

## 4. Discussion

Phages offer a natural and effective alternative to antibiotics for the treatment of bacterial infections. However, to most efficiently utilize phage therapeutics, phage-host interactions need to be understood. No studies to date have extensively investigated the initial interactions of *C. difficile* phages to their hosts. Here, we present the first data on the adsorption and irreversible binding of three *C. difficile* phages with the view to gaining insights into their infectivity on ten clinically relevant *C. difficile* strains. Comparing the adsorption dynamics to phage host range is an important first step to understanding the barriers to successful infection and subsequent lysis of bacteria by phages.

Phages CDHM1, CDHM3 and CDHM6 could only lytically infect two of the ten *C. difficile* strains tested. On the remaining eight strains either no infection or partial and turbid clearing was observed and the latter is likely due to the phages being temperate [15]. The host range of CDHM1, CDHM3 and CDHM6 is very similar to *C. difficile* myoviruses in our collection CDKM15 and CDKM9 [21], this could suggest the phages may be all binding to the same bacterial surface receptor. The receptor for the three phages used in this study has not yet been identified but it has been hypothesized the phages could be binding to the S-layer of *C. difficile*, which is a paracrystaline layer that surrounds the bacterial cells and varies between ribotypes [50].

The phages bound by different adsorption efficiencies to all strains and adsorption was phage-host specific. The adsorption profile of CDHM1 was very different to CDHM3 and CDHM6 but similar to the two other *C. difficile* phages tested in this study: CDHM2 and CDHM5. For this group of phages it was consistently observed that high percentages of phage bound to strains they could lytically infect and the phages bound by a lower percentage to strains they do not infect [25]. This pattern is consistent with other *C. difficile* phages described in the literature, such as phages phi41, phi56 and phiCD140 that bind by ~70, 99 and 90% respectively to their propagation hosts after 30 min [25,51]. In comparison CDHM3 only bound by a high adsorption percentage to its propagation host but poorly to ATJ, which it infects and CDHM6 bound by low percentages to all strains regardless of whether the phage was able to infect the strain or not. The adsorption profiles of CDHM3 and CDHM6 highlight infection did not necessarily correlate with high adsorption efficiency. Similarly phage phiHISC binds to its host *Listonella pelagia* by less than 5% after 26 min but still is able to infect and replicate within the host [52].

To understand the different adsorption patterns in the three phages, the annotated putative tail fiber proteins were compared on the grounds that they provide the initial contact to their host and are therefore responsible for phage adsorption [29]. The differences in putative tail fiber proteins is in part reflected in the variances seen between the adsorption profiles of CDHM1 versus CDHM3 and CDHM6, whose putative tail fiber protein sequences shared 100% homology. However, this is not the full story as there were clear differences in the adsorption profiles of CDHM3 and CDHM6. The results highlight there is no functional link between putative tail fiber sequences and experimental findings, but this is the first time it has been investigated for *C. difficile* phages. Our data will add to the growing field of trying to establish what determines a successful infection.

Interestingly, the putative tail fiber protein sequences of CDHM3 and CDHM6 were also longer in comparison to CDHM1. It could be possible the extra amino acids present in their tail fibers help to facilitate less specific, reversible adsorption to strains they are not able to infect, which was observed in this study. Previous studies have highlighted long tail fibers are responsible for reversible binding, for example for phage T4 its long tail fibers are responsible for reversible binding to *E. coli* and short tail fibers for irreversible binding [30]. Similarly more than one tail fiber may could be involved in phage binding to *C. difficile* cells [51,53]. Other studies have shown several different phage proteins are likely to be involved in phage adsorption, such as receptor-degrading enzymes, which can degrade the bacterial cell surface to facilitate phage adsorption and are part of the phages’ tail structure [54]. One major group of depolymerases are hydrolases and multiple putative cell wall hydrolases proteins have been identified in the genomes of phages CDHM1, CDHM3 and CDHM6. Another protein identified to play a role in adsorption is the tailspike protein, which was recently shown to be involved in instant phage binding followed by a gradual release [55]. However, tailspike proteins have not been identified in the phages used in this study but similar proteins could be present within the phages.

Other contributing factors to the differences observed in the adsorption percentages could be attributed to changes in the expression of phage receptors on the bacterial cells as not all strains continuously express their phage receptor at a constant rate [47,54,55]. Furthermore, bacteria can mask the surface of their phage receptor, as was observed with the *S. aureus* phage where the bacteria produced a cell wall anchored virulence factor, immunoglobulin G-binding protein A that blocked and significantly reduced phage adsorption [56]. Similar mechanisms could be in play for *C. difficile* phages CDHM1, CDHM3 and CDHM6 but can only be confirmed once the phage receptor has been identified.

The adsorption data showed that the phages bind to strains they could not infect, which could suggest that phage receptors on the tested *C. difficile* cells are conserved, but infection is blocked by downstream processes. To understand why this was the case the proportion of phages that bound irreversibly was determined and the data highlighted there was a clear relationship between irreversible phage binding and infection for all three phages, which was also observed for CDHM2 and CDHM5. The general trend was that on strains not infected by the phages, only a small proportion of phages irreversibly bound and most molecules ~70 to 94% bound reversibly and could be broken by vortexing. The lack of irreversible binding could explain why phage infection was not established. On strains where the phages produced turbid clearing ~50% irreversible binding occurred, which suggests that phages can bind and thus likely eject their DNA into these strains but they cannot replicate efficiently. On strains infected by the phages, over 85% irreversible phage binding was observed regardless of whether total phage adsorption was 75% or 30%. This suggests even minimal adsorption is sufficient for infection if a high proportion of these molecules are irreversibly bound. As there is a clear relationship between irreversible binding and infection it could suggest phages are binding to another receptor on the bacterial surface for irreversible binding. This has also been shown for phages that infect *Bacillus subtilis*, who use one receptor for reversible binding and another for irreversible binding [40,41].

The adsorption dynamics of the phages to common hospital pathogens were also investigated to *E. coli*, *P. aeruginosa* and MRSA strains as they may be found in similar environments as *C. difficile*. All three phages bound poorly to these strains, which further highlights phages are specific for *C. difficile*. Similar observations were made for another *C. difficile* phage phiCD140, which only bound by 1% to *C. perfrigens*, *C. Sordellii*, *C. bifermentans* and *Lactobacillus spp* [25].

To conclude this is the first study to extensively analyze adsorption dynamics of *C. difficile* phages, to determine if poor adsorption to strains impacts host range. The data has shown that low proportions of phage adsorption does not necessarily equate to no phage infection but over 80% of irreversible binding is needed for infection. This study has also shown there is no distinct relationship between infection, adsorption and putative phage tail fiber sequence for *C. difficile* myoviruses. In future work we will identify the phage receptors on the bacterial host, characterize other phage proteins that could be involved in phage binding and define the steps post phage binding that may limit infection. This work has provided valuable insights into *C. difficile* phage-host interactions that are both of fundamental interest and can help with future strategies to designing optimal phage therapeutic cocktails.

## 5. Patents

The phages described are part of a Leicester patent, pending. European Patent Application No. 13759275.4 and US Patent Application No. 14/423284.

## Figures and Tables

**Table 1:** Host range and efficiency of plating of *C. difficile* phages CDHM1, CDHM3 and CDHM6.

*C. difficile* Ribotype	Strain Identity	Infectivity of Phages ^1^			Efficiency of Plating (%)		
		CDHM1	CDHM3	CDHM6	CDHM1	CDHM3	CDHM6
013	ASH	−	+	−	0	0	0
014/020	ATJ	++	++	++	100	73	81
026	ARU	+	−	+	0	0	0
027	CD105LC1	−	+	−	0	0	0
076	CD105HE1	++	++	++	100	100	100

^1^ No infection is presented as −; hazy and turbid clearing by + and infection by ++.

**Table 2:** Percentage of irreversibly bound phages calculated from the total number of phages bound to different clinically relevant *C. difficile* ribotypes.

Ribotype	Strain Identity	Irreversible Binding (%) of *C. difficile* Phages ^1^		
		CDHM1	CDHM3	CDHM6
002	AKC	13	4	14
005	AIN	18	12	15
013	ASH	3	51	21
014/020	ATJ	90	93	90
026	ARU	59	2	58
027	CD105LC1	7	52	5
076	CD105HE1	95	88	97
078	ASS	16	9	7
087	APT	29	8	10
107	ARZ	9	12	7

^1^ Average irreversible phage binding was calculated from three independent experiments each with three technical repeats.

**Table 3:** The percentage of phages CDHM2 and CDHM5 that adsorbed and irreversibly bound to different clinically relevant *C. difficile* ribotypes.

Ribotype	Strain Identity	CDHM2		CDHM5	
		Adsorption (%) ^1^	Irreversible Binding (%) ^1^	Adsorption (%) ^1^	Irreversible Binding (%) ^1^
002	AKC	22	29	33	29
005	AIN	11	6	10	12
013	ASH	3	11	13	9
014/020	ATJ	82	92	82	97
026	ARU	32	2	10	15
027	CD105LC1	36	13	29	21
076	CD105HE1	79	98	80	97
078	ASS	20	30	17	20
087	APT	17	8	10	30
107	ARZ	26	36	17	14

^1^ Average adsorption and irreversible phage binding percentages were calculated from three independent experiments each with three technical repeats.

## Supplementary Materials

The following are available online at http://www.mdpi.com/1999-4915/10/8/411/s1, Table S1: A list of *C. difficile* phages used for phylogenetic analyses of their tail fibers and their accession numbers, Figure S1: The effect of salts on *C. difficile* phage adsorption and Figure S2: Adsorption and irreversible binding of *C. difficile* phages (a) CDHM1, (b) CDHM2, (c) CDHM3, (d) CDHM5 and (e) CDHM6 to ten clinical strains of *C. difficile* that are representatives of 10 different ribotypes.
