# Supplementary material for: Unravelling the Links between Phage Adsorption and Successful Infection in Clostridium difficile
Source: Viruses. 2018 Aug 6;10(8):411. doi: 10.3390/v10080411 (PMC6116197; doi:10.3390/v10080411)
Supplement: Supplementary file 1 [file viruses-10-00411-s001.pdf]

Supplementary materials

Supplementary Table S1. A list of *C. difficile* phages used for phylogenetic analyses of their tail fibres and their accession numbers.

C. difficile **Phage**	**Taxonomy**	**Tail fiber protein accession number**
CDKM15	Myoviridae	ANT45172.1
CDKM9	Myoviridae	ANT45092.1
CDHM1	Myoviridae	YP_009032171.1
CDHM3	Myoviridae	MH256665
CDHM6	Myoviridae	MH256666
phiC2	Myoviridae	YP_001110744.1
CD27	Myoviridae	YP_002290901.1
phiCD481-1	Myoviridae	CEK40592.1
phiCD505	Myoviridae	CEK40649.1
phiCD506	Myoviridae	CEK40722.1
phiCDHM11	Myoviridae	CDL68837.1
phiCDHM13	Myoviridae	CDL65303.1
phiCDHM14	Myoviridae	CDU85306.1
phiCDHM19	Myoviridae	CDW17212.1
phiMMP01	Myoviridae	CEK40783.1
phiMMP02	Myoviridae	YP_006990503.1
phiMMP03	Myoviridae	CEK40864.1
phiMMP04	Myoviridae	YP_006990576.1

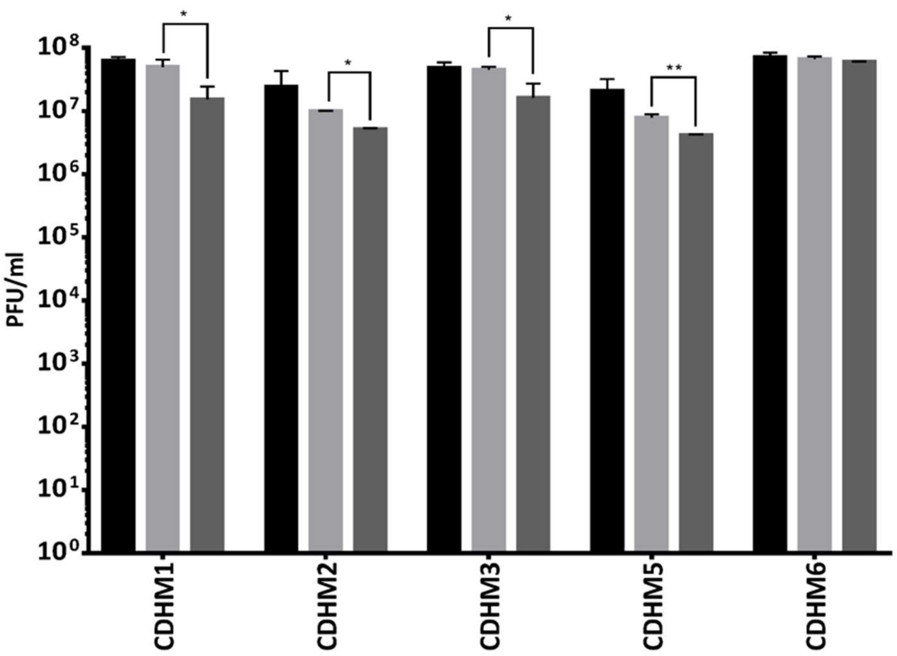

Supplementary Figure S1. The effect of salts on *C. difficile* phage adsorption. Adsorption assays of phages CDHM1, CDHM2, CDHM3, CDHM5 and CDHM6 were conducted to their propagation host CD105HE1 either in the absence of salts (light grey bars) or in presence of salts (dark grey) and compared to the original phage titre (black bars). Plotted are titres of free phage particles calculated from average titres from three independent repeats each with three technical replicates and error bars represent standard error of the mean. Statistically significant differences between free phage titres when no salts versus salts are added have been highlighted ($p \leq 0.05$ (*) and $P \leq 0.01$ (**)).

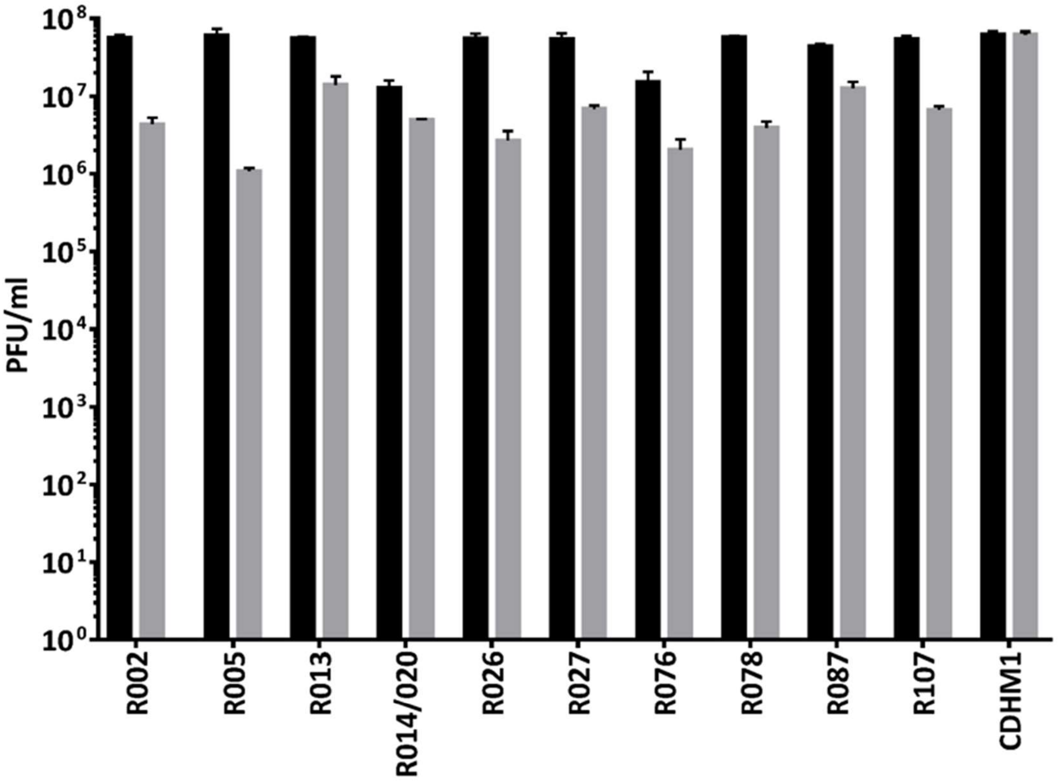

PFU/ml
10^8
10^7
10^6
10^5
10^4
10^3
10^2
10^1
10^0
R002
R005
R013
R014/020
R026
R027
R076
R078
R087
R107
CDHM1

(a)

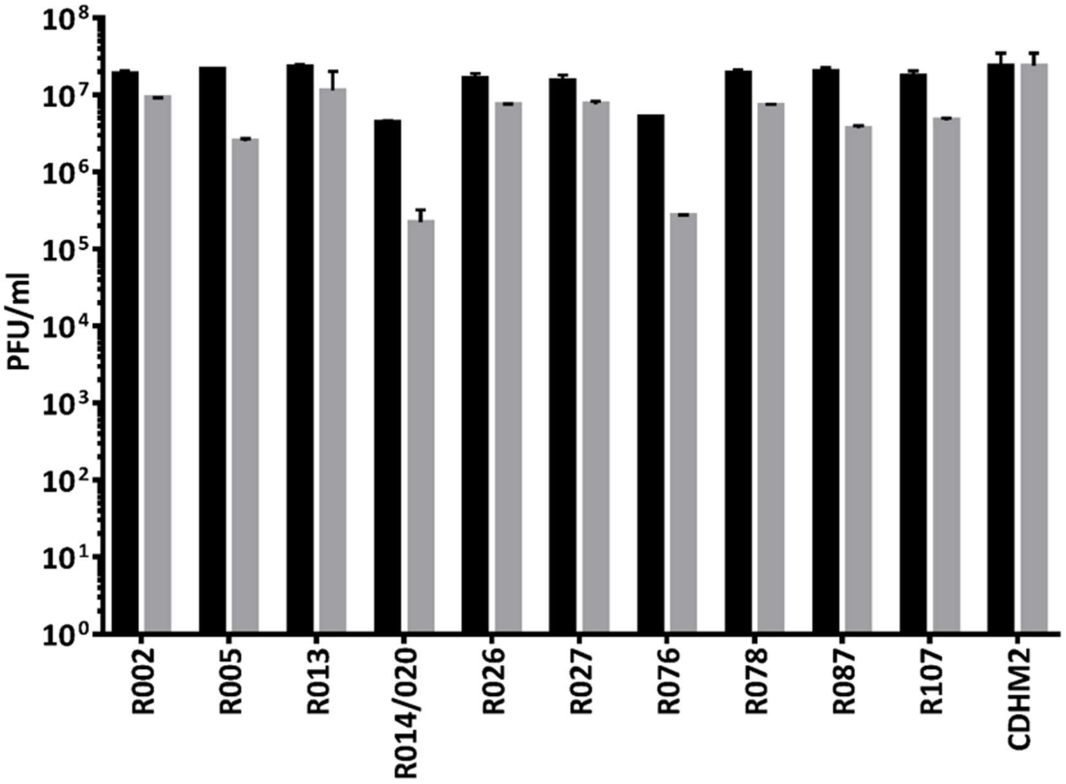

PFU/ml
10^8
10^7
10^6
10^5
10^4
10^3
10^2
10^1
10^0
R002
R005
R013
R014/020
R026
R027
R076
R078
R087
R107
CDHM2

(b)

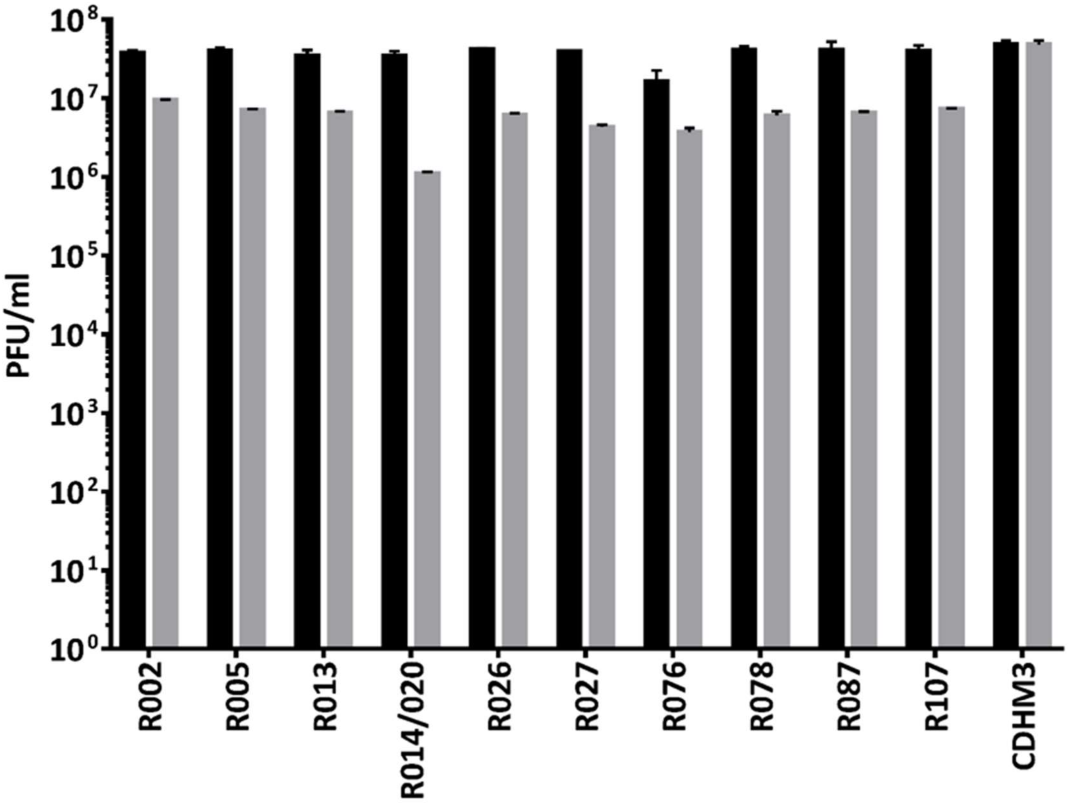
PFU/ml
10^8
10^7
10^6
10^5
10^4
10^3
10^2
10^1
10^0
R002
R005
R013
R014/020
R026
R027
R076
R078
R087
R107
CDHM3

(c)

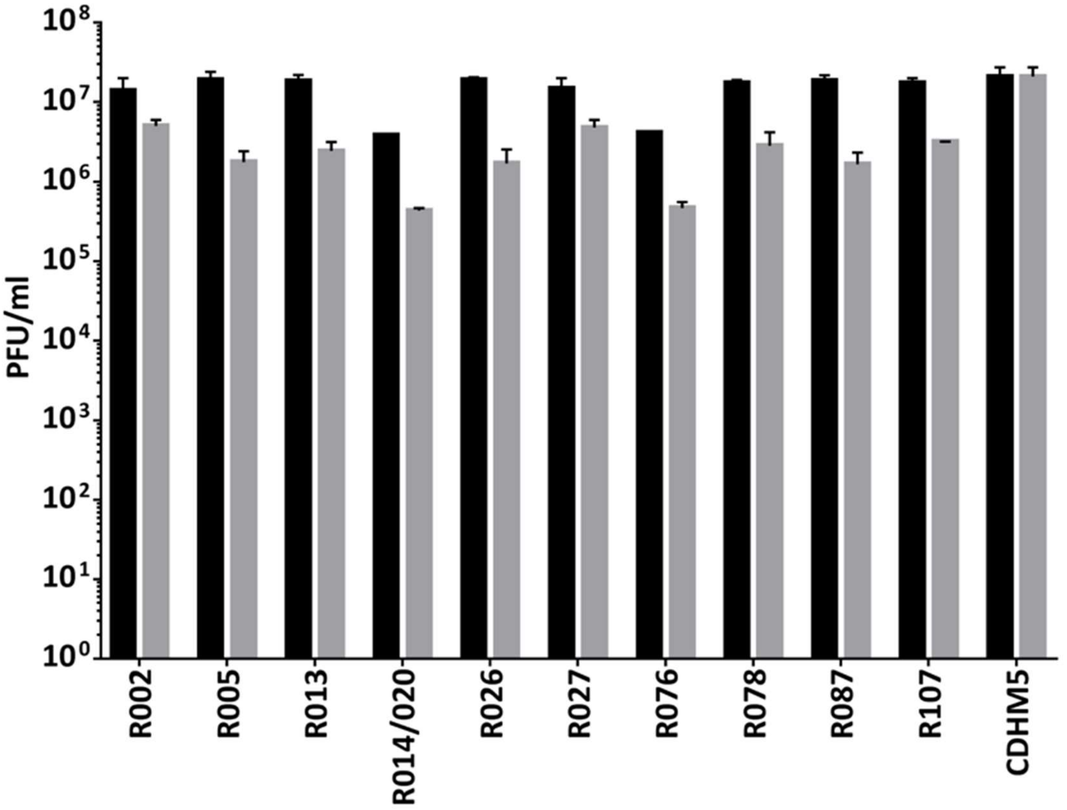
PFU/ml
10^8
10^7
10^6
10^5
10^4
10^3
10^2
10^1
10^0
R002
R005
R013
R014/020
R026
R027
R076
R078
R087
R107
CDHM5

(d)

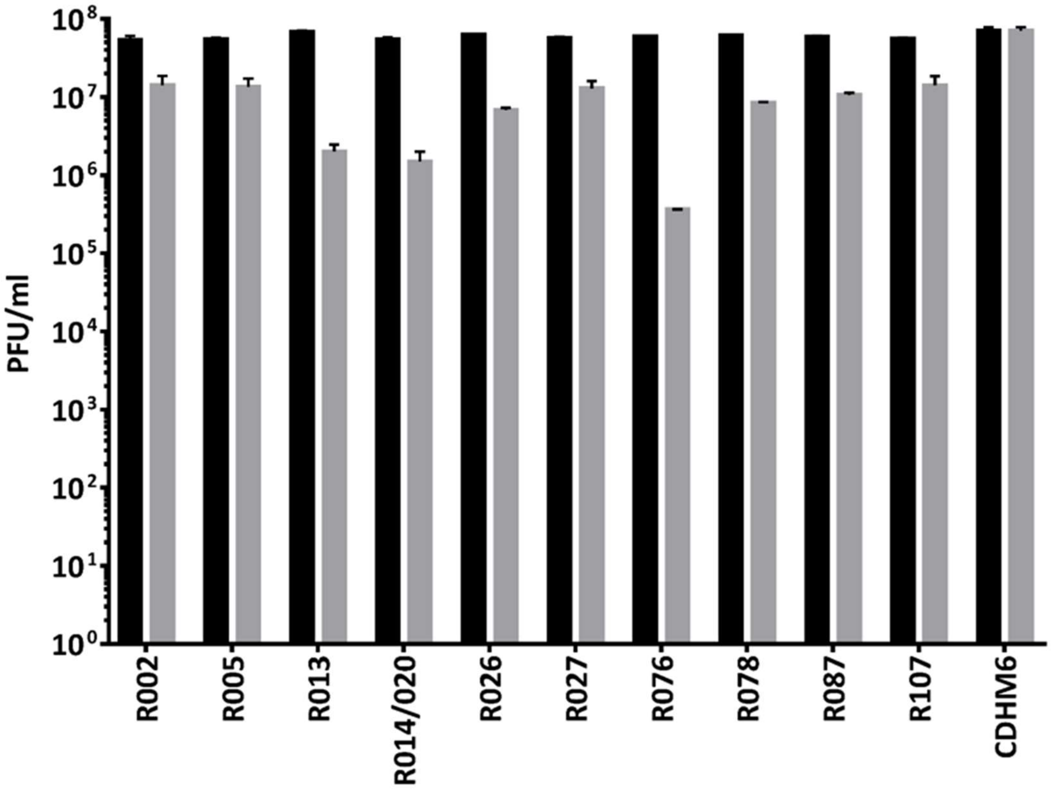

(e)

Supplementary Figure S2. Adsorption and irreversible binding of *C. difficile* phages (a) CDHM1, (b) CDHM2, (c) CDHM3, (d) CDHM5 and (e) CDHM6 to ten clinical strains of *C. difficile* that are representatives of 10 different ribotypes. Black bars represent titre of unabsorbed phages (S1) and light grey bars show titres of reversibly bound phages (S2). Both S1 and S2 values were used to calculate total adsorbed phages and irreversibly bound phages and equations used are detailed in the methods section. A phage only control in buffer was included and their phage titres are included in all graphs. All bars represent average phage titres from three independent experiments, each with three technical repeats and error bars represent standard error of the mean.
